# Fine-scale heterogeneity in *Schistosoma mansoni* force of infection measured through antibody response

**DOI:** 10.1073/pnas.2008951117

**Published:** 2020-08-31

**Authors:** Benjamin F. Arnold, Henry Kanyi, Sammy M. Njenga, Fredrick O. Rawago, Jeffrey W. Priest, W. Evan Secor, Patrick J. Lammie, Kimberly Y. Won, Maurice R. Odiere

**Affiliations:** ^a^Francis I. Proctor Foundation, University of California, San Francisco, CA 94143;; ^b^Department of Ophthalmology, University of California, San Francisco, CA 94143;; ^c^Eastern and Southern Africa Centre of International Parasite Control, Kenya Medical Research Institute, Nairobi, Kenya;; ^d^Center for Global Health Research, Kenya Medical Research Institute, Kisumu, Kenya;; ^e^Division of Foodborne, Waterborne and Environmental Diseases, Centers for Disease Control and Prevention, Atlanta, GA 30333;; ^f^Division of Parasitic Diseases and Malaria, Centers for Disease Control and Prevention, Atlanta, GA 30333;; ^g^Neglected Tropical Disease Support Center, Task Force for Global Health, Decatur, GA 30030

**Keywords:** schistosomiasis, parasitology, antibodies, epidemiology, infectious disease transmission

## Abstract

Schistosomiasis is among the most common parasitic diseases in the world, with over 142 million people infected in low- and middle-income countries. Measuring population-level transmission is centrally important in guiding schistosomiasis control programs. Traditionally, human *Schistosoma mansoni* infections have been detected using stool microscopy, which is logistically difficult at program scale and has low sensitivity when people have low infection burdens. We compared serological measures of transmission based on antibody response to *S. mansoni* soluble egg antigen (SEA) with stool-based measures of infection among 3,663 preschool-age children in an area endemic for *S. mansoni* in western Kenya. We estimated force of infection among children using the seroconversion rate and examined how it varied geographically and by age. At the community level, serological measures of transmission aligned with stool-based measures of infection (ρ = 0.94), and serological measures provided more resolution for between-community differences at lower levels of infection. Force of infection showed a clear gradient of transmission with distance from Lake Victoria, with 94% of infections and 93% of seropositive children in communities <1.5 km from the lake. Force of infection increased through age 3 y, by which time 65% (95% CI: 53%, 75%) of children were SEA positive in high-transmission communities—2 y before they would be reached by school-based deworming programs. Our results show that serologic surveillance platforms represent an important opportunity to guide and monitor schistosomiasis control programs, and that in high-transmission settings preschool-age children represent a key population missed by school-based deworming programs.

## Significance

Schistosomiasis is one of the most common parasitic diseases in the world, and most infected people (90%) live in Africa. Global control efforts use measures of population-level transmission to target programs and assess progress toward elimination. Monitoring *Schistosoma mansoni* transmission has traditionally relied on examining stool with microscopy, which is difficult to scale in large programs and has low sensitivity as infection burdens decline. Our results show that antibody-based measures of transmission align well with stool-based measures, provide higher sensitivity at lower levels of transmission, and enable fine-scale estimates of force of infection by geography and age. The findings represent a major step toward use of serosurveillance to guide schistosomiasis control efforts in Africa.

Schistosomiasis is one of the most common parasitic diseases in the world, with an estimated 142 million people infected globally, over 90% of whom live in sub-Saharan Africa (1). The disease is caused by infection with blood flukes of the genus *Schistosoma* spp. *S. hematobium* (causing urogenital infections), and *Schistosoma mansoni* (causing gastrointestinal infections) account for the majority of infections in humans. Adult *S. mansoni* worms live in the mesenteric veins adjacent to the human intestinal tract. *S. mansoni* is highly endemic in the Lake Victoria region, where the environment is conducive to support transmission. Freshwater snails—*S. mansoni*’s obligate intermediate host— thrive in the lake’s ecosystem. The primary route of transmission to humans is dermal contact with the parasite’s free-swimming stage (cercariae) in surface waters (2).

The World Health Organization has targeted the global elimination of schistosomiasis as a public health problem by 2025, defined as a reduction to <1% prevalence of heavy intensity infections across sentinel sites (1). A cornerstone of the global control strategy has been delivery of the drug praziquantel to school-age children (5 to 15 y) through mass drug administration (MDA) campaigns in endemic regions (3). School-age children have been a focus of MDA efforts because *S. mansoni* infection intensity and egg shedding peaks at ages 10 to 15 y (2), and because schools provide a logistically convenient approach for locating children through large-scale programs. Measuring *S. mansoni* infection among children is crucially important to target program efforts to populations with ongoing transmission and to monitor control program progress. Monitoring transmission requires sensitive markers of infection that can be measured in large-scale surveys.

Seroepidemiologic studies that measure transmission intensity through antibodies in blood have made valuable contributions to the study of transmission dynamics and burden of infectious diseases such as malaria (4, 5) and arboviruses (6–9). Serological testing is growing in use to monitor progress of many neglected tropical disease control programs such as trachoma (10), lymphatic filariasis (11), and onchocerciasis (12). The near-term potential for integrated, population-based serologic surveillance with multiplex assays creates an opportunity for more frequent, broader monitoring of diseases with well-validated serological assays and methods to translate antibody response into measures of transmission (13, 14). To our knowledge, there has been no detailed assessment of seroepidemiologic methods used to estimate transmission based on *S. mansoni* antibody measurements, nor has there been a thorough comparison of serological measures of transmission against the current standard of stool-based measures of patent infection.

Our objective was to estimate *S. mansoni* transmission through antibody responses measured among 3,663 preschool-age children living in 30 communities in western Kenya near Lake Victoria, an area endemic for *S. mansoni*. We studied the relationship between serologic measures and stool-based measures of transmission intensity and used serology to create high-resolution assessments of force of infection among children by distance from Lake Victoria and by age. Our results show that population-based, serological surveys create an important opportunity to monitor *S. mansoni* transmission and control program progress in endemic settings and further suggest that preschool-age children represent an important sentinel population for serologic monitoring in higher transmission settings.

## Results

### Study Population and Setting.

The analysis included 3,663 children ages 2 mo to 5.5 y who were originally enrolled from 30 communities that participated in a cluster randomized controlled trial to measure the effect of community-wide versus school-based mass distribution of praziquantel to reduce *S. mansoni* infection in Mbita, western Kenya (15). Communities were selected within 5 km of Lake Victoria from among those with ≥25% *S. mansoni* prevalence that had not received mass treatment with praziquantel (16). Preschool-age children ≤5 y were assessed annually from 2012 to 2014 in repeated cross-sectional surveys that collected blood and stool specimens, and samples were tested for the presence of infection (in stool) and for antibodies to *S. mansoni* soluble egg antigen (SEA) (*SI Appendix*, Fig. S1). The area is highly endemic for malaria and schistosomiasis infection.

**Fig. 1.**
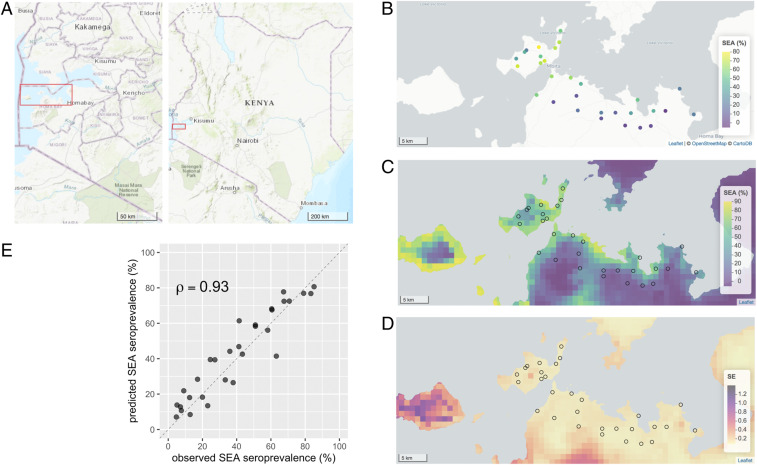
Spatial heterogeneity of antibody response to *Schistosoma* *mansoni* SEA antigen in 30 study communities near Mbita, Kenya, 2012 to 2014. (*A*) Overview of the study location. Red rectangles mark the extent of the remaining map panels. (*B*) SEA seroprevalence in the 30 study communities measured from 3,663 preschool-age children. (*C*) Predicted seroprevalence at 1-km resolution from a geostatistical model. (*D*) Approximate SEs of the predicted proportion SEA seropositive from the geostatistical model. (*E*) Geostatistical model predicted SEA seroprevalence versus observed for the 30 study communities in 2014. Spearman rank correlation (ρ) estimate between predicted and observed. The diagonal line is 1:1. Created with notebook: https://osf.io/wu2gx/.

*S. mansoni* infection prevalence decreased slightly over the study period (Table 1), but was not significantly different by treatment arms (15), so we combined data from all years (2012 to 2014) in the present analysis. The average number of children measured per community during the 3-y study period was 122 (median: 117; interquartile range: 84, 163; range: 59, 196). *S. mansoni* infection and seroprevalence increased with age, consistent with endemic transmission (Table 1). Environmental covariates were strongly correlated with seroprevalence and infection (*SI Appendix*, Fig. S2). Model-based geostatistical predictions of seroprevalence as a function of location plus a range of environmental covariates showed that infection was highest close to Lake Victoria, with some heterogeneity over the study area (Fig. 1). Predicted prevalence of stool-based infection showed a similar pattern and was consistent with geospatial analyses of infection among school-age children in the region (*SI Appendix*, Fig. S3) (17).

**Table 1.** Number of samples tested and *Schistosoma mansoni* prevalence by SEA and Kato-Katz, stratified by age and by year, Mbita, Kenya, 2012 to 2014

	SEA seroprevalence			Kato-Katz prevalence		
	*N* samples	*N* positive	%	*N* samples	*N* positive	%
Overall	3,663	1,749	48	3,426	869	25
Age, years completed						
<1 y	51	12	24	47	5	11
1 y	570	132	23	503	68	14
2 y	780	295	38	714	150	21
3 y	890	483	54	844	211	25
4 y	1,214	727	60	1,166	384	33
5 y	158	100	63	152	51	34
Year						
2012	1,120	557	50	1,072	297	28
2013	1,187	595	50	1,172	308	26
2014	1,356	597	44	1,182	264	22

Created with notebook: https://osf.io/k7drz/. SEA, soluble egg antigen.

### Comparison of Serological and Stool-Based Measures of Transmission.

**Fig. 2.**
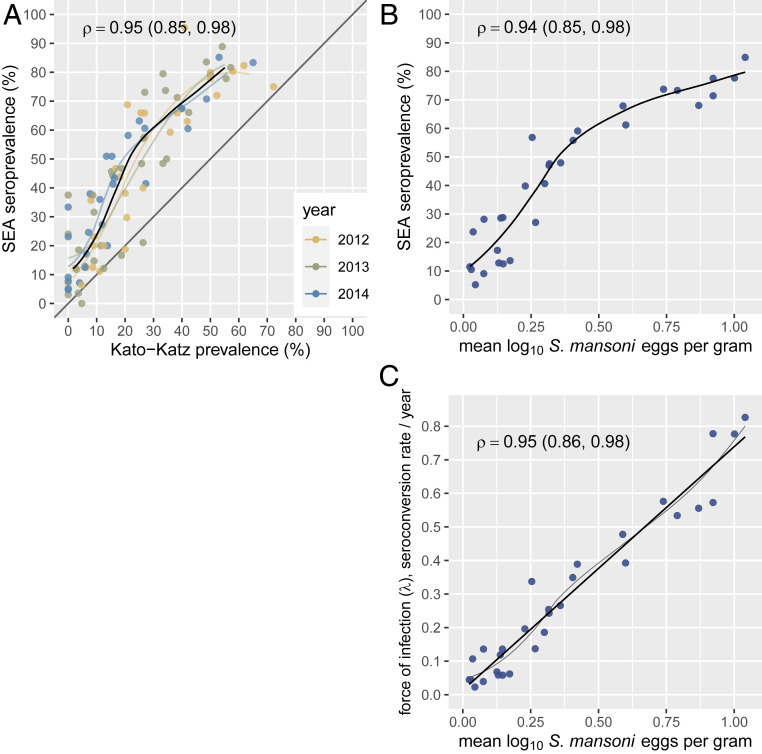
Community-level seroprevalence of *Schistosoma mansoni* SEA response and infection measured by double-slide Kato-Katz stool microscopy in Mbita, Kenya, 2012 to 2014. (*A*) Relationship between SEA seroprevalence and Kato-Katz prevalence in the 30 study communities over a 3-y period. Locally weighted regression fits are provided for each year, along with a single fit from measurements pooled across years (heavy line). Regression fits trimmed to 95% of the data to reduce edge effects. (*B*) Community-level *S. mansoni* SEA seroprevalence as a function of mean log_10_ *S. mansoni* eggs per gram of stool, a conventional measure of infection burden. (*C*) Community-level *S. mansoni* force of infection estimated using SEA seroconversion rate from a proportional hazards model as a function of mean log_10_ *S. mansoni* eggs per gram of stool. Panels include locally weighted regression fits; the force of infection panel also includes a linear fit (heavy line). Each panel includes Spearman rank correlation estimates (ρ) and their bootstrapped 95% CI. Created with notebook: https://osf.io/3wzfv/.

Community-level seroprevalence was consistently higher than infection prevalence detected by stool examination, but measurements were highly correlated (Spearman’s ρ = 0.95) and the relationship was consistent across study years (Fig. 2*A*). At the community level, we observed a wider range of seroprevalence at low levels of stool-based prevalence, suggesting that serology had higher sensitivity and better resolution compared with stool-based testing at lower levels of transmission. Community-level seroprevalence and infection prevalence were relatively stable over the 3-y study period, with high correlation between years (*SI Appendix*, Fig. S4) even in the context of ongoing MDA. Preschool-age children were not directly targeted in the study’s MDA campaigns, but treatment with praziquantel was offered for all study children with positive stool microscopy.

At the community level, serological measures of transmission intensity aligned closely with stool-based measures of infection intensity. We measured a child’s *S. mansoni* infection intensity using eggs per gram of stool, which, at the population level, has been used as a measure of parasite burden (18). We also estimated each community’s average force of infection with the seroconversion rate among children (details in *Materials and Methods*). There was a monotonic relationship between community mean infection intensity and seroprevalence, and a linear relationship between mean infection intensity and force of infection (Fig. 2 *B* and *C*).

**Fig. 3.**
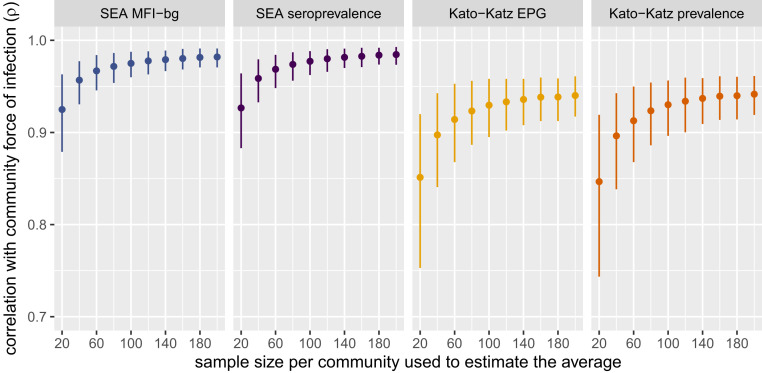
Spearman rank correlation between measures of *Schistosoma mansoni* and community-level serological force of infection (λ) estimated with different simulated sample sizes per community. Force of infection was measured by the seroconversion rate in the full sample (median sample size: 117 measurements per community). Estimates and error bars mark bootstrapped means and 95% CIs. In each replicate, samples of different sizes were drawn with replacement from each of 30 communities before calculating the means and correlation with force of infection estimated in the full sample. EPG, eggs per gram of stool; MFI-bg, median fluorescence intensity minus background on the Luminex platform; SEA, soluble egg antigen. Created with notebook: https://osf.io/3wzfv/.

Simulations that varied the sample size per community between 20 and 200 measurements showed that the correlation remained strong between summary measures of SEA or Kato-Katz prevalence and community-level force of infection estimated in the full sample (Fig. 3). In the present setting, we estimated serological surveys that measure as few as 20 to 40 children per community could reliably rank communities according to their underlying transmission intensity (ρ > 0.9), similar to estimates for dengue seroprevalence in Bangladesh (7).

### Gradient of Transmission with Distance from Lake Victoria.

**Fig. 4.**
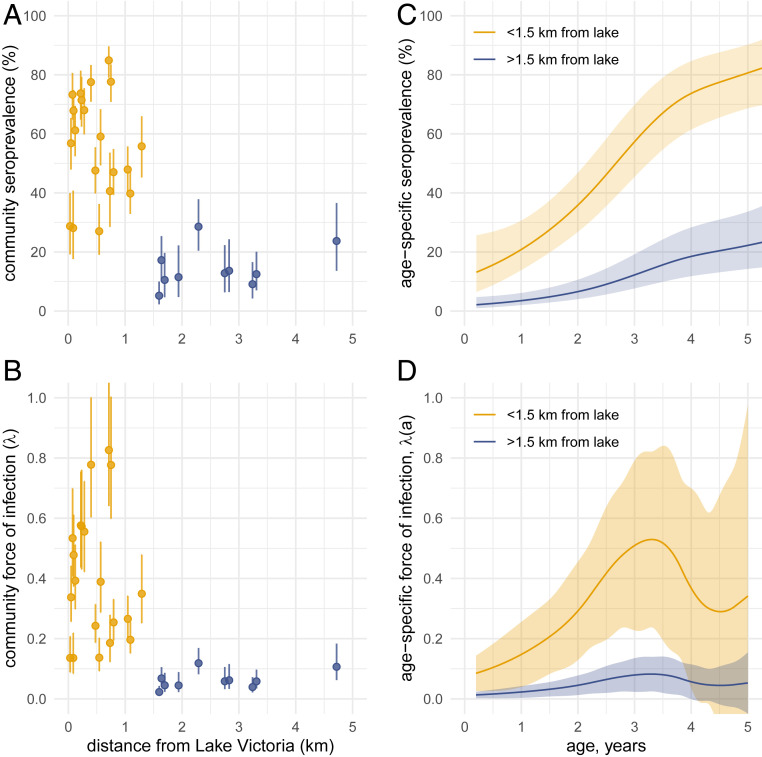
*Schistosoma mansoni* SEA seroprevalence and force of infection among preschool-age children by distance to Lake Victoria and age in Mbita, Kenya, 2012 to 2014. (*A*) Relationship between distance from Lake Victoria and community-level SEA seroprevalence in the 30 study communities over the 3-y period. Vertical bars mark exact 95% binomial CIs. (*B*) Relationship between distance from Lake Victoria and community-level force of infection estimated by the SEA seroconversion rate in the 30 study communities. Vertical bars on community-level force of infection estimates mark 95% CIs estimated from a semiparametric proportional hazards model. In *A* and *B*, points are colored by communities further than 1.5 km from the lake, used to examine differences in age-varying force of infection. (*C*) Age-dependent seroprevalence estimated with cubic splines, stratified by distance to Lake Victoria. (*D*) Age-dependent force of infection derived from seroprevalence curves in *C*. Shaded regions in *C* and *D* indicate approximate simultaneous 95% CIs. Created with notebooks: https://osf.io/fnxs7/ and https://osf.io/dnckx/.

Based on studies using stool examination only, we hypothesized that serological measures of transmission intensity would be higher among children living closer to Lake Victoria compared with children living further away from the lake (17, 19). *S. mansoni* is transmitted to humans through dermal exposure to cercariae in surface water and the predominant exposure in this setting was Lake Victoria. We summarized community-level seroprevalence and force of infection by distance from the lake and found a clear gradient of transmission (Fig. 4 *A* and *B*). Among study children, 94% of infections by Kato-Katz and 93% of SEA seropositive preschool-age children lived within 1.5 km of Lake Victoria, consistent with highly focal transmission.

### Age-Varying Force of Infection.

We hypothesized that age-varying force of infection would be higher among children who lived closer to Lake Victoria compared with children who lived further away. We also hypothesized that higher transmission would be reflected in a steeper age–seroprevalence curve that plateaued at a higher level, consistent with seroepidemiologic patterns observed across diverse pathogens (4, 20) but which has not been previously examined for *S. mansoni*. We stratified communities by those that were within 1.5 km of Lake Victoria versus those further away based on the observed distribution of community-level force of infection, noting that all communities with high seroprevalence and force of infection were <1.5 km from Lake Victoria (not a prespecified cutoff). We estimated age-varying seroprevalence and force of infection using semiparametric cubic splines that controlled for child age between groups.

Higher transmission intensity among communities close to Lake Victoria was reflected in a steeper age–seroprevalence curve (Fig. 4*C*). By 5 y of age, 80% of children living in communities <1.5 km from Lake Victoria had serological evidence of infection, compared with 20% of children living further from the lake. Overall, the hazard of seroconversion was 6.5 times higher (95% CI, 4.2, 10.3) among preschool-age children living <1.5 km from Lake Victoria compared to children living further away. Age-varying force of infection estimated from age-structured seroprevalence with semiparametric cubic splines showed that force of infection increased through the third year of age (Fig. 4*D*). At age 3 y, the seroconversion rate among children living <1.5 km from Lake Victoria was 0.5 per child-year, which means that among 3-y-old children who were not previously infected, 50% were infected before age 4 y. A sensitivity analysis by quintiles of distance from the lake showed that there were only two distinct transmission profiles that were delineated by the 1.5-km threshold (*SI Appendix*, Fig. S5).

## Discussion

This study represents a comprehensive examination of *S. mansoni* seroepidemiology among children living in endemic settings. Close alignment between measures of transmission intensity derived from stool infections and antibody response provides evidence to support the use of well-characterized schistosome antigens to monitor transmission and burden of schistosomiasis in similar settings. In this endemic population, antibody response to *S. mansoni* SEA provided information about transmission at fine spatial scales and at high resolution across ages. All high transmission communities fell within 1.5 km of Lake Victoria, and in this study 94% of infections among preschool-age children fell within this narrow band around the lake. Our estimates of age-specific force of infection suggest that infection pressure begins very early and that force of primary infection peaks by age 3 to 4 y among children living in communities close to the lake.

Our study supports a growing consensus for the need to expand control programs to treat and prevent infection among preschool-age children in endemic settings (21, 22). In communities along the shores of Lake Victoria, antibody response to SEA showed that 55% of children were infected by age 3 y, at least 2 y before they would be eligible to receive treatment through school-based MDA. Global control programs currently exclude preschool-age children due in part to lack of evidence for efficacy and safety in this group, but clinical trials are in progress to develop safe delivery methods for praziquantel for children under 6 y of age (23, 24). Complementary strategies that focus on environmental interventions should be promoted in tandem with MDA to help reduce transmission among this vulnerable age group (25).

Some key epidemiologic insights presented here have been established through stool microscopy. For example, model-based geostatistical maps of SEA seroprevalence (Fig. 1) and Kato-Katz prevalence (*SI Appendix*, Fig. S3) were overall very similar. Studies throughout the Lake Victoria region have documented a gradient of infection among school-age children with distance from the lakeshore, with higher infection prevalence closer to the lake (16, 17, 19, 26–28). The mechanism driving the distance–infection relationship is thought to be the predominance of *S. mansoni*’s intermediate snail hosts in lakeshore habitat and frequency of human water contact (26). Close agreement in epidemiologic inference between stool and serological methods confirms the validity of using serology to monitor schistosomiasis.

Serological monitoring among older, school-age children could be less useful in high-transmission settings if SEA IgG remains high in infected individuals and decays slowly after treatment, even in the absence of reinfection. Most studies that have followed IgG response longitudinally enrolled older individuals and found that IgG remains elevated for many months or years after treatment with praziquantel (summarized in ref. 29). In this study, >80% of children were seropositive by age 5 y in communities close to the lake. If SEA IgG levels remain elevated for multiple years following infection, then a majority of school-age children will be seropositive and changes in transmission would only be detectable among children born into lower transmission conditions. Longitudinal measurement of antibody levels to SEA among children born in the same setting in western Kenya as the present study showed maternal IgG clearance by age 20 wk and no evidence for antibody waning among a limited number of children infected through age 24 mo (30). Longer, longitudinal follow-up of children who have been successfully treated and not reinfected would help confirm SEA IgG decay rates, but clearly delineated, wide separation between SEA antibody responses at low and high levels observed in this study (*SI Appendix*, Fig. S1) provides indirect evidence for a robust and durable IgG response with little waning among preschool-age children. The relationship between community-level infection prevalence and seroprevalence was consistent across years in the presence of annual chemotherapy (Fig. 2*A*), and suggests seroprevalence among preschool-age children provides a robust, complementary strategy to Kato-Katz testing for monitoring population endemicity.

Our results suggest two reasons to consider the use of serology as a complement to stool-based testing to monitor schistosomiasis. First, IgG response integrates exposure over time, thus providing more information about a child’s past exposure than current infection. This benefit was most evident in communities with lower levels of infection (Kato-Katz prevalence, <25%; Fig. 2*A*). Of 52 community surveys with Kato-Katz prevalence <25%, 10 had zero positives—in those same surveys SEA seroprevalence ranged from 3 to 38%. The additional resolution serology provides compared with stool-based infection could prove valuable to control programs as populations approach elimination by providing a more refined picture of transmission from a cross-sectional community survey. Second, seroepidemiologic models can estimate force of infection from a cross-sectional, age-structured serological survey. Stool collection is arguably more difficult than dried blood spot collection and measuring force of infection through reinfection rates in stool requires longitudinal monitoring—both considerations increase the cost of surveillance.

A shift in age–seroprevalence curves for *S. mansoni* with reductions in transmission closely aligns with patterns observed for many pathogens (20), and aligns with the “peak shift” of infection intensity observed for schistosomiasis and many other parasitic diseases (31, 32). That *S. mansoni* SEA exhibits this classic seroepidemiologic pattern supports the use of modeling approaches originally developed in the context of vaccine-preventable diseases and arboviruses to study transmission through cross-sectional serological surveys (33). The use of widely generalizable modeling approaches to estimate *S. mansoni* transmission in cross-sectional, serological surveys further supports its inclusion in integrated surveillance platforms, which benefit from shared analysis methods to translate antibody response into measures of transmission (14). Multiplex antibody testing from a lymphatic filariasis surveillance survey in coastal Kenya recently identified communities with a clear signature of *S. mansoni* transmission based on SEA seroprevalence (34), The methods we have used herein, such as model-based geostatistical mapping and force of infection estimation, could further extend analysis outputs of integrated serosurveillance platforms to help inform control programs.

In addition to broader surveillance, serology could directly inform the design and evaluation of schistosomiasis intervention studies. For example, some communities near Lake Victoria have higher forces of transmission and are less responsive to MDA than others. Heterogeneity in transmission and responsiveness to intervention emerges as early as 2 y after control program initiation (35, 36). Our results suggest that a single cross-sectional serological survey before programs start could estimate community-level seroprevalence or force of infection and provide guidance for which communities warrant more intensive intervention. Furthermore, antibody responses among preschool-age children may be useful to reflect the impact of an intervention, thereby improving monitoring and evaluation.

The multiplex antibody assay used to measure SEA response in this study is not commercially available, and there is currently no rapid diagnostic test based on SEA. At this time, large-scale monitoring of SEA response is therefore most viable through integrated serologic surveillance with specimens tested in multiplex in larger reference laboratories, such as the Kenya Medical Research Institute (KEMRI) laboratory in Nairobi where the present samples were analyzed. This poses a challenge for the implementation of antibody-based monitoring for schistosomiasis programs, particularly ahead of the 2025 World Health Organization goal for elimination of schistosomiasis as a public health problem. Nevertheless, serological surveys with multiplex antibody testing across a range of pathogens are becoming more common (14). Broader testing of specimens for schistosomiasis SEA in surveys conducted by other programs could provide additional information to complement schistosomiasis-specific monitoring activities (34), which currently rely on single-pathogen microscopy or circulating cathodic antigen tests (37).

This analysis had limitations. The 30 study communities were located over a relatively small area (277 km^2^). We were unable to test whether spatial predictions of *S. mansoni* seroprevalence remain accurate if sampling locations are more widely distributed over larger areas, such as districts or nations. Fine-scale heterogeneity in force of infection observed in this study suggests that program monitoring at the level of districts might be too coarse as populations approach elimination, but surveys with high spatial resolution over larger geographic areas could provide confirmatory results. Strong relationships between remotely sensed covariates and measures of *S. mansoni* infection observed in this study (*SI Appendix*, Fig. S2) suggest it might be possible to use geospatial models to predict seroprevalence over larger areas with less densely measured communities, but it remains an open question. It might also be possible to improve geostatistical model predictions by extending models to include more geospatial layers (17) or by including more refined measures of snail habitat derived from remotely sensed images, such as those recently proposed in Senegal (38). The force of infection analyses assumed no seroreversion over the age range (change from seropositive to seronegative), and longitudinal studies of children following infection and successful treatment without reinfection would be useful to confirm postinfection SEA IgG kinetics. Finally, since SEA IgG appears to be a durable response, it cannot detect reinfection or superinfections; thus, force of infection estimates based on SEA provide a lower bound of the true transmission intensity.

In conclusion, antibody response to *S. mansoni* SEA antigen among preschool-age children enabled fine-scale estimates of transmission heterogeneity in an endemic setting in western Kenya. Serological estimates aligned with those from stool-based testing and provided higher resolution about between-community heterogeneity in infection than microscopy at lower levels of transmission. Our results support the inclusion of *S. mansoni* in population-based serologic surveillance platforms to target control programs and monitor their effectiveness.

## Materials and Methods

### Ethics Statement.

The study protocol was reviewed and approved by ethical review committees at the KEMRI (SSC number 2185) and the US Centers for Disease Control and Prevention (protocol 6249). The analysis protocol was further reviewed and approved by the ethical review committee at the University of California, San Francisco (protocol 19-28772). The study was explained to participants and field staff obtained written, informed consent at the time of enrollment in each study year. Parents or guardians provided consent for the preschool-age children enrolled.

### Study Design.

Thirty villages were enrolled in a community-based, randomized, and controlled trial that delivered annual praziquantel MDA to primary school children (school-based delivery, 15 communities) or community-wide (all people 5 y or older, 15 communities). Communities that had *S. mansoni* prevalence of ≥25% prevalence by Kato-Katz in an earlier survey of the study region (16) were selected to participate in the trial. At the time of the study, there were no guidelines for the inclusion of preschool-age children in schistosomiasis MDA programs, so only preschool-age children identified as positive for *S. mansoni* infection by Kato-Katz were treated with crushed praziquantel under medical supervision. Additional details of the trial and study population have been previously reported (15). Measurement followed a repeated cross-sectional design. All children aged 1 to 5 y were eligible for study inclusion and were enrolled in separate cross-sectional surveys in May to July of 2012, 2013, and 2014. Some children might have been measured repeatedly across years, but the study did not track children longitudinally. A small number of children between ages 2 mo and 1 y (*n* = 51) and slightly older than 5 y (*n* = 158) were enrolled outside of the target age range and were included in the present analysis.

### Specimen Collection and Testing.

Eligible children provided stool and blood at a central location in each community. Plastic stool containers were given to the parents, and an attempt was made to collect a single stool sample from the child at each community’s central monitoring location. Stool samples were transported in cool boxes with ice packs to the Ministry of Health’s Division of Vector-Borne Diseases (DVBD) laboratory in Homabay, where they were processed the same day. From each stool sample, two slides were prepared by the Kato-Katz method and examined by independent, trained microscopists for the presence of *S. mansoni* eggs. Arithmetic means of the two slides were calculated and expressed as eggs per gram (EPG). The average EPG values from the two readers were used in the analysis.

Blood from a fingerstick was collected into a serum capillary collection tube (∼100 μL) and transported to the DVBD laboratory in Homabay for centrifugation. Sera were stored at −20 °C in the DVBD laboratory and were transported to the KEMRI Neglected Tropical Diseases laboratory in Kisumu, where they were stored at −80 °C until sent to KEMRI/Eastern and Southern Africa Centre of International Parasite Control (ESACIPAC) laboratory in Nairobi. Specimens were analyzed on the Luminex platform (Bio-Plex 200) for antibody levels to the *S. mansoni* SEA and recombinant Sm25 antigen, an integral glycoprotein found in microsomal preparations of *S. mansoni* adult worms (GenBank accession number M37004.1) (15). Details of the assay preparation have been previously reported (15). Samples were tested in duplicate and those with a coefficient of variation of >15% between duplicate wells for greater than three positive antibody responses were repeated.

In this analysis, we focused on SEA antibody responses because Sm25 responses did not clearly distinguish two subpopulations of seronegative and seropositive (infected or previously infected) children (*SI Appendix*, Fig. S1). A SEA seropositivity cutoff was determined through a receiver operating characteristic curve analysis of known positive and negative sera with a cutoff for the KEMRI/ESACIPAC instrument determined as 965 MFI-bg (sensitivity, 97.5%; specificity, 100%) as previously described (15).

### Prevalence Mapped with Model-Based Geostatistics.

For each location in the study region, we calculated the distance from Lake Victoria using the global surface water layer (39), and obtained average monthly minimum temperatures and average monthly precipitation from WorldClim (40). We considered including elevation in the geostatistical model but omitted it because it was highly correlated with distance from the lake (*SI Appendix*, Fig. S2). We modeled prevalence as a function of distance to the lake, temperature, and precipitation using a spatial generalized additive model with binomial error structure and cubic splines for each covariate. The model included a spatially structured, community-level random effect with a low rank Gaussian Process and Matern covariance structure, implemented in the R mgcv package (41). We made predictions at 1-km resolution (minimum scale of the WorldClim data) and summarized observed versus predicted prevalence at the 30 community locations.

### Community-Level Measures of Infection.

We estimated community-level prevalence of *S. mansoni* infection as the proportion of children with detected eggs in stool. We estimated community mean eggs per gram on the log_10_ scale because infection intensities were highly skewed. For children without any eggs detected in stool, we recoded them as 1 to enable the log_10_ transformation before estimating community means. Thus, mean eggs per gram is reported as the overall community average infection intensity, not the average infection intensity among infected children. We compared community level estimates of seroprevalence, force of infection (seroconversion rate, described below), Kato-Katz prevalence, and mean eggs per gram of stool using bivariate scatter plots with locally weighted regression smooths and Spearman rank correlation. We estimated the Spearman rank correlation of community-level seroprevalence across study years to assess the stability of estimates over time. We did not adjust community-level seroprevalence or infection prevalence for age because the age distribution was very similar across all communities (*SI Appendix*, Fig. S6).

We conducted a simulation study to assess the effect of community-level sample size on the relationship between multiple SEA and Kato-Katz measures and community-level force of infection measured by the SEA seroconversion rate. The objective of the simulation was to determine whether a small number of measurements in a community could still provide rank-order information about community-level force of infection. From the empirical measurements, we resampled children with replacement from each community using a nonparametric bootstrap with community-level sample sizes that varied from 20 to 200 measurements per community. In each bootstrap replicate, we estimated the mean log_10_ SEA MFI-bg, SEA seroprevalence, mean log_10_ Kato-Katz eggs per gram, and Kato-Katz prevalence, and then estimated the Spearman rank correlation between each measure and community-level force of infection measured by the seroconversion rate in the full sample. We used the 2.5% and 97.5% percentiles of the bootstrap distribution to estimate 95% CIs for correlation estimates at each sample size.

### Age-Structured Seroprevalence and Force of Infection.

We used a current status, semiparametric proportional hazards model to estimate force of infection from age-structured seroprevalence (42). We fit a generalized additive mixed model with binomial error structure and complementary log-log link:

$$\begin{array}{l} \log-\log\left[1-P\left(Y_{ij}=1|A_{ij},D_{i},\;S_{ij},b_{i}\right)\right]=g\left(A_{ij}\right)+\beta_{1}I\left(D_{i}<1.5\right)+\\ \beta_{2}I\left(S_{ij}=2013\right)+\beta_{3}I\left(S_{ij}=2014\right)+b_{i}, \end{array}$$ [1]

where $Y_{ij}$ is SEA seropositivity, $A_{ij}$ is age, and $S_{ij}$ is sampling year for child *j* in community *i*. $D_{i}$ is distance from Lake Victoria to community *i*. Community-level random effects, $b_{i}$, were included to allow for correlated outcomes within community. Function g(⋅) was parameterized with cubic splines that had smoothing parameters chosen through cross-validation. An indicator was included for communities within 1.5 km of Lake Victoria versus those further away based on the observed distribution of community-level seroprevalence, noting that all communities with high seroprevalence were <1.5 km from Lake Victoria. Since this distance cutoff was not prespecified, we conducted a sensitivity analysis that stratified communities by quintiles of distance and found that there were only two age–seroprevalence profiles that were captured by the 1.5-km cutoff (*SI Appendix*, Fig. S5). We selected the final model compared to a constant rate model without year fixed effects using Akaike information criterion (AIC) and Bayesian information criterion (BIC) (*SI Appendix*, Table S1 includes detailed specifications and AIC/BIC values). We estimated the hazard ratio of seroconversion associated with living <1.5 km from the lake as ${\hat{\theta }}_{HR}=\exp\left({\hat{\beta }}_{1}\right)$.

We estimated age-specific seroprevalence from the model as follows:

$$\hat{P}\left(Y=1|A=a,D=d,S=s\right)=1-\text{exp}\left(-\exp\left[\hat{\eta }\left(a\right)\right]\right),$$ [2]

and we estimated age-specific force of infection from the model as follows:

$$\hat{\lambda }\left(a\right)=\eta^{'}\left(a\right)\exp\left[\hat{\eta }\left(a\right)\right],$$ [3]

where $\eta^{'}\left(a\right)$ is the first derivative of the linear predictor from the complementary log-log model, η(a) (43). We estimated $\eta^{'}\left(a\right)$ using finite differences from the model predictions (41, 42). We estimated approximate, simultaneous 95% CIs around age-specific seroprevalence and age-specific force of infection curves with a parametric bootstrap (10,000 replicates) from posterior estimates of the model parameter covariance matrix (44).

### Community-Level Force of Infection by Distance from Lake Victoria.

We estimated community-level force of infection from the age-structured seroprevalence model (Eq. **1**). To estimate the marginal average force of infection in each community from ages 1 through 5 y, we integrated over age. If F(a) is the seroprevalence at age a, the definition of the hazard (force of infection) from current status data is $\lambda \left(a\right)=F^{'}\left(a\right)/F\left(a\right)$ and the cumulative hazard from birth is $\int_{0}^{a}\lambda \left(a\right)da=-\text{log}\left[1-F\left(a\right)\right]$. The average hazard from ages $a_{1}$ to $a_{2}$ is thus as follows:

$$\lambda =\int_{a_{1}}^{a_{2}}\lambda \left(a\right)da=\frac{\log\left[1-F\left(a_{1}\right)\right]-\log\left[1-F\left(a_{2}\right)\right]}{a_{2}-a_{1}}.$$ [4]

We substituted F(a) with predictions from Eq. **2** allowing means to vary according to the model’s estimated community-level random effects. We estimated 95% CIs on community-level force of infection estimates using a parametric bootstrap (10,000 replicates) from posterior estimates of the model parameter covariance matrix (44).

## Supplementary Material

Supplementary File

## Data Availability

Data and replication files required to conduct the analyses are available through the Open Science Framework (https://osf.io/rnme8/) and Dryad (https://doi.org/10.7272/Q6DZ06J3). Analyses used R statistical software, version 4.0.2. Community location data required to replicate the geostatistical model fits are not publicly available to protect participant confidentiality but are available from the corresponding author upon request, pending appropriate human subjects review and approval. Anonymized data (de-identified individual participant data) have been deposited in Open Science Framework (https://osf.io/rnme8/) and Dryad (https://doi.org/10.7272/Q6DZ06J3) (45).
