# Supplementary material for: Fine-scale heterogeneity in Schistosoma mansoni force of infection measured through antibody response
Source: Proc Natl Acad Sci U S A. 2020 Aug 31;117(37):23174–81. doi: 10.1073/pnas.2008951117 (PMC7502727; doi:10.1073/pnas.2008951117)
Supplement: Supplementary File [file pnas.2008951117.sapp.pdf]

Fine-scale heterogeneity in *Schistosoma mansoni* force of infection measured through antibody response

Arnold et al. *Proc Natl Acad Sci U.S.A.* 2020.

Supplementary Appendix

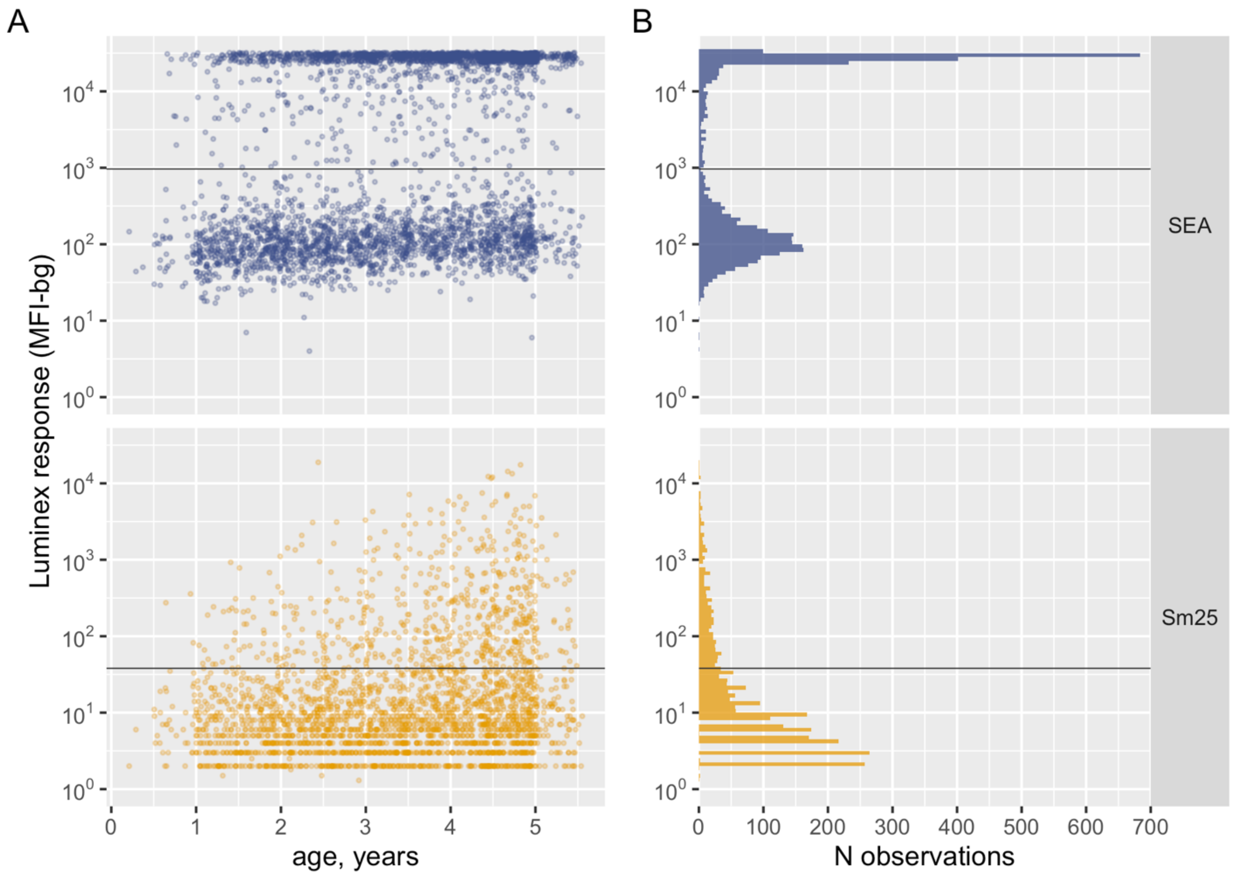

Fig. S1 Antibody responses to *Schisotosoma mansoni* SEA and Sm25 antigens from 3,663 preschool aged children near Lake Victoria, Mbita, Kenya, 2012–2014. (**A**) Luminex response in by age. (**B**) Distribution of Luminex responses. In both panels, the horizontal line marks the seropositivity cutoff derived through receiver operating characteristic curve analyses of known positive and presumed negative samples (SEA: 965 MFI-bg, sensitivity = 97.5%, specificity = 100%; Sm25: 38 MFI-bg, sensitivity = 93.5%, specificity = 97.3%) (15). MFI-bg: median fluorescence intensity minus background. Created with notebook: https://osf.io/b47ek/ .

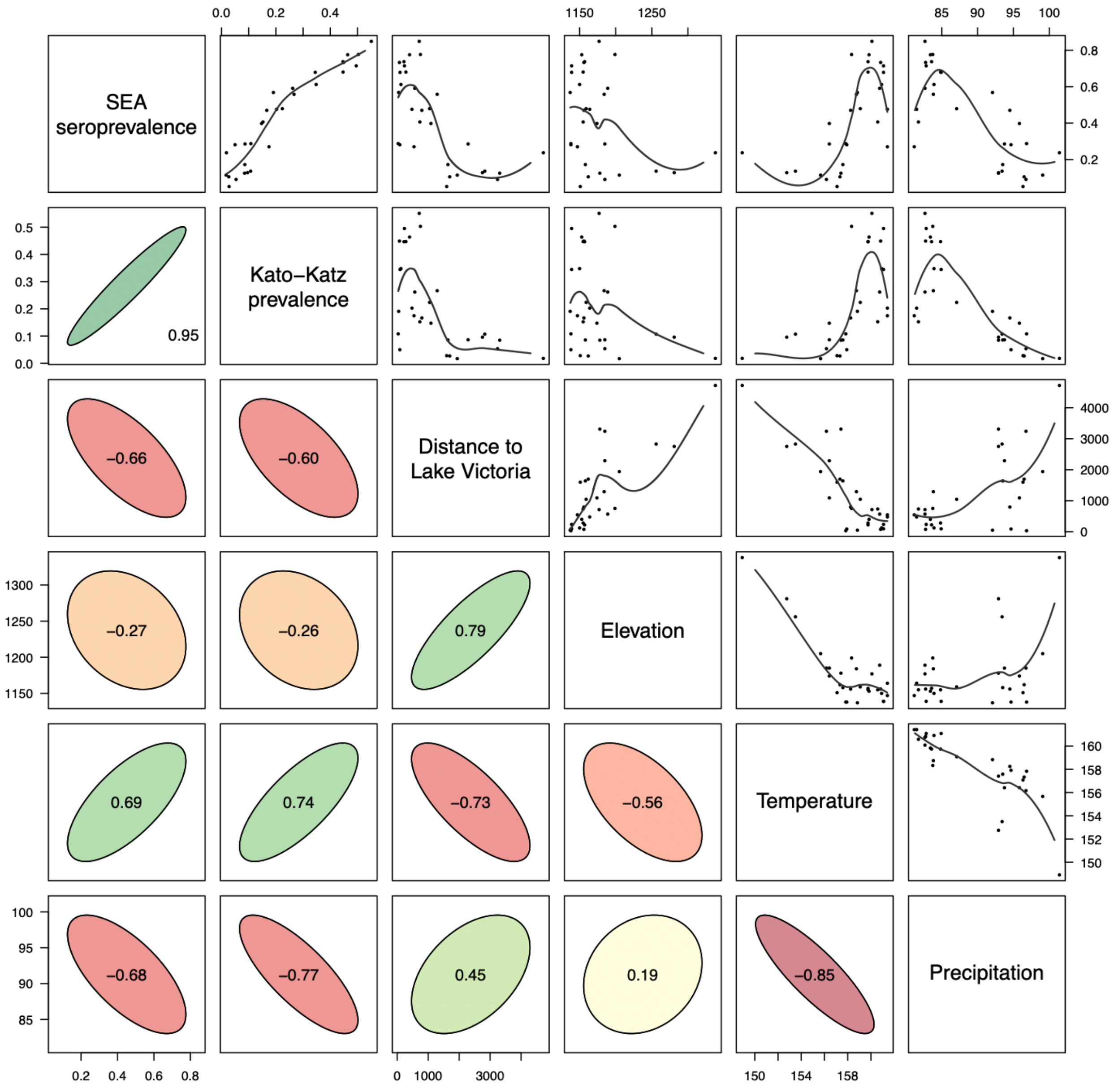

Fig. S2 Bivariate pairs plot of *Schisotosoma mansoni* SEA seroprevalence, Kato–Katz prevalence, and environmental covariates in 30 communities near Lake Victoria, Mbita, Kenya, 2012–2014. Scatter plots include nonparametric locally weighted regression fits trimmed to reduce edge effects. Correlation ellipses depict the strength of the association on the basis of the Spearman rank correlation (printed). Preschool aged children < 5 years old were measured for SEA seroprevalence (N = 3,663) and Kato–Katz prevalence (N = 3,426). Created with notebook: https://osf.io/wu2gx/.

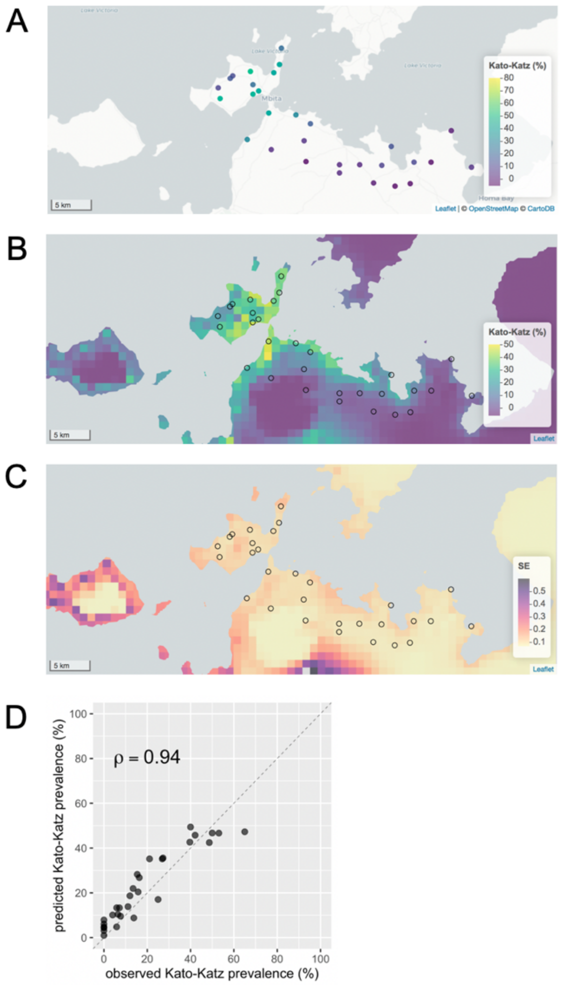

Fig. S3 Spatial heterogeneity of *Schisotosoma mansoni* infection prevalence in Mbita, Kenya, 2012–2014. Infection measured using dual slide Kato-Katz microscopy in 3,426 stool specimens among pre-school aged children. (**A**) Infection prevalence in the 30 study communities. (**B**) Predicted prevalence at 1 km resolution from a geostatistical model. (**C**) Approximate standard errors of the predicted proportion infected from the geostatistical model. (**D**) Predicted versus observed infection prevalence for the 30 study villages in 2014 and spearman rank correlation (ρ). The diagonal line is 1:1. Created with notebook: https://osf.io/wu2gx/.

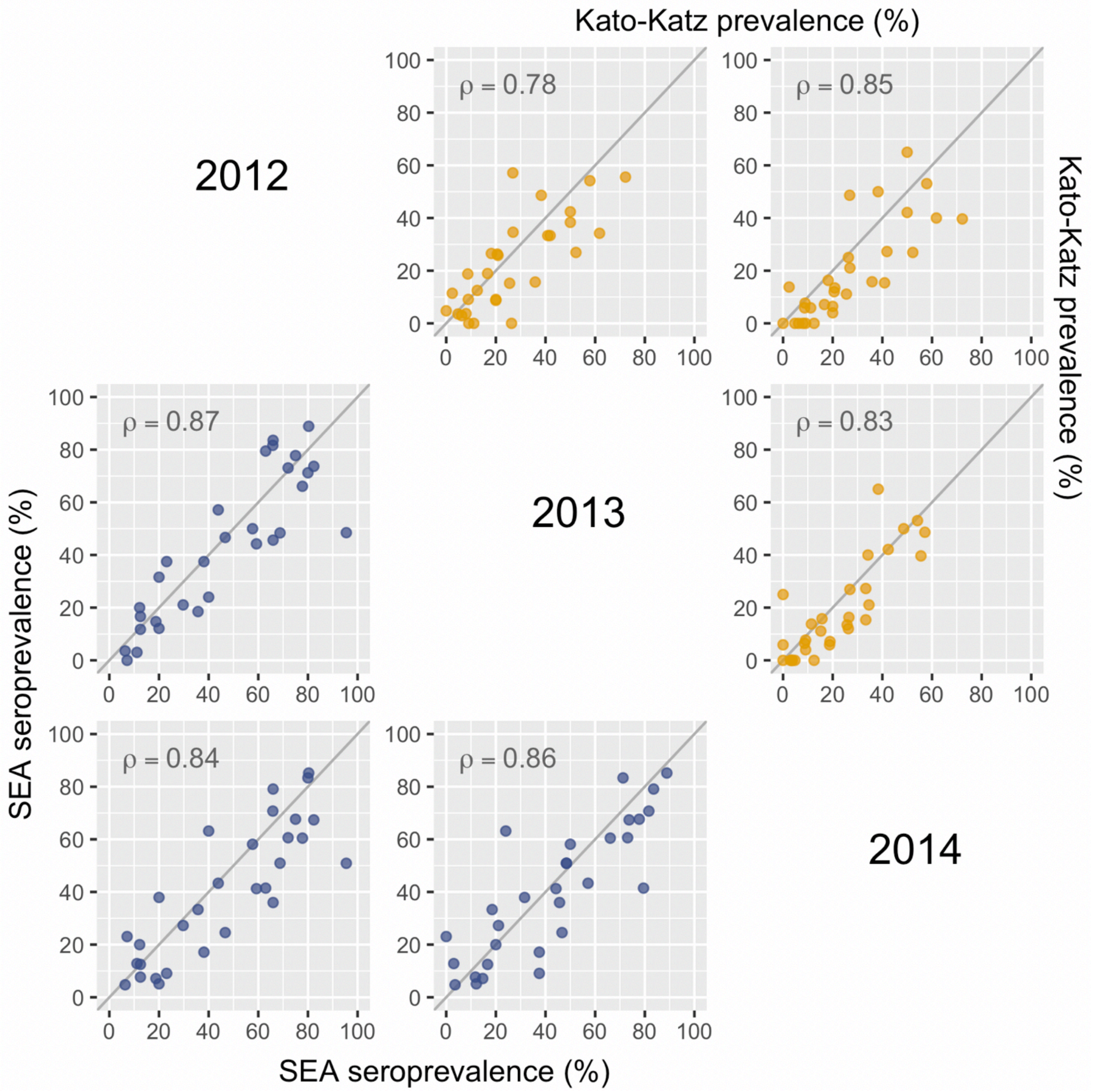

Fig. S4 Relationship between community level SEA seroprevalence and Kato-Katz infection prevalence across years. Bivariate pairs plot of SEA seroprevalence (lower panels) and Kato-Katz infection prevalence (upper panels) by year in the 30 study communities, including Spearman rank correlation estimates (ρ). Each panel shows the relationship between community level prevalence between years on the diagonal. Created with notebook: https://osf.io/3wzfv/ .

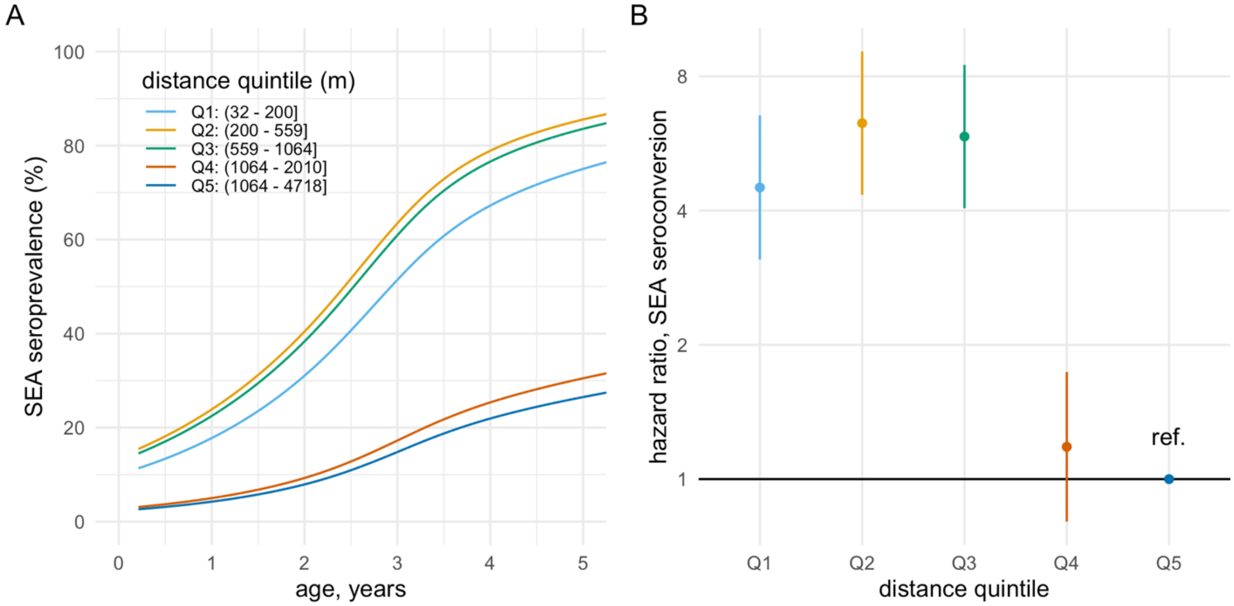

Fig. S5 *Schisotosoma mansoni* SEA by age and quintile of distance from Lake Victoria, Mbita, Kenya, 2012–2014. (**A**) Age-dependent seroprevalence estimated using semiparametric cubic splines as in the primary analysis, but allowing the relationship to vary by quintile of distance from Lake Victoria. Each distance quintile includes measurements from six communities (*n*=3,663 measurements from 30 villages). (**B**) Hazard ratio of seroconversion among children in each distance quintile estimated from the semiparametric model, with children in the fifth quintile as reference (ref.). Created with notebook: https://osf.io/dnckx/.

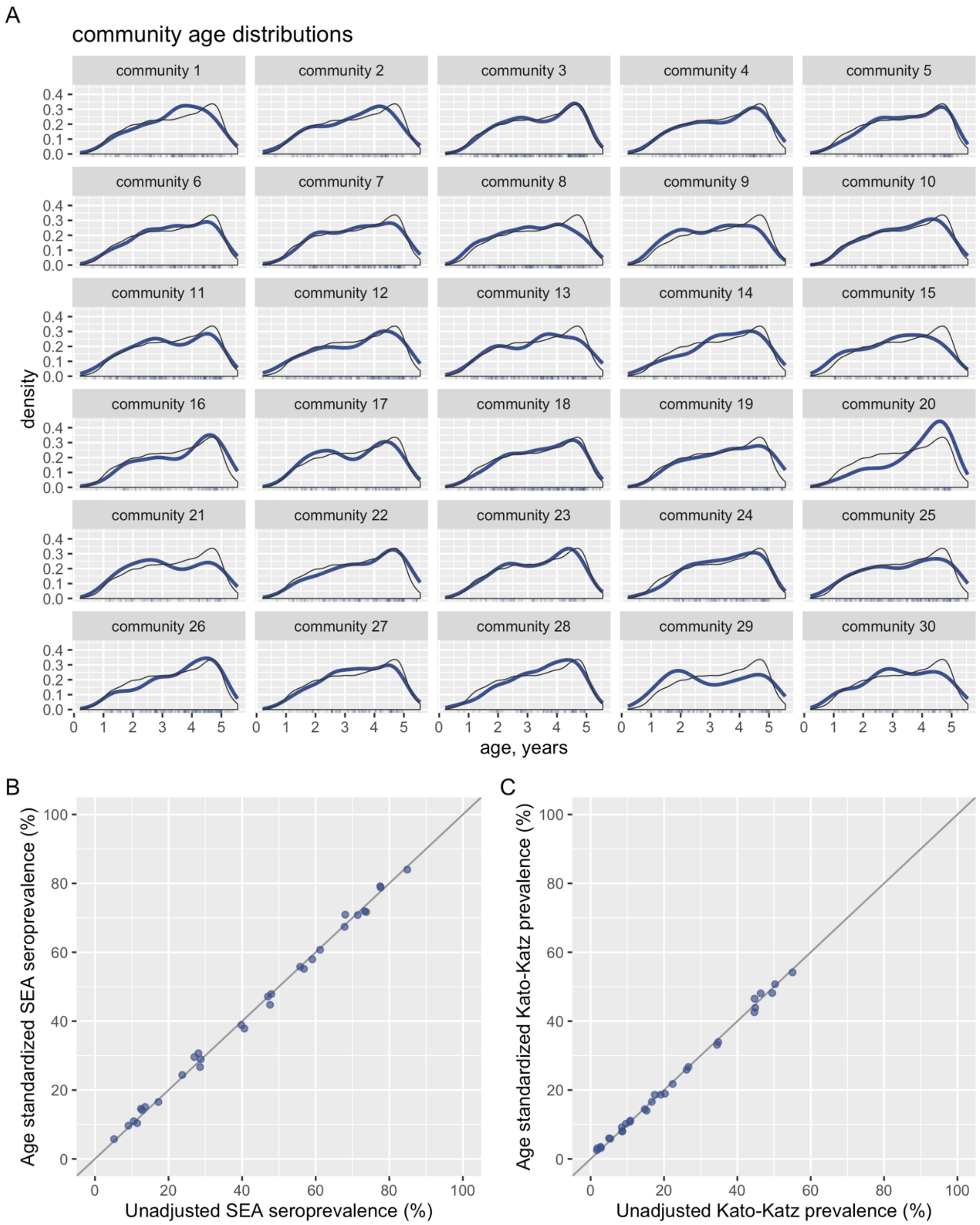

Fig. S6 Age distribution and effect of age adjustment on community level prevalence estimates based on 3,663 samples collected in 30 communities near Lake Victoria, Mbita, Kenya, 2012–2014. (**A**) Age distribution in each community. The heavy line is each community's age distribution, the light grey line marks the age distribution across all communities. (**B**) Age standardized community level seroprevalence to *Schistosoma mansoni* SEA antigen versus unadjusted seroprevalence; the diagonal line is 1:1. (**C**) Age standardized community level infection prevalence to *S. mansoni* measured with dual slide Kato-Katz microscopy versus unadjusted prevalence; the diagonal line is 1:1. Created with notebook: https://osf.io/fnxs7/ .

Table S1. Summary of current status survival model specifications, AIC, and BIC values. Model 4 was used in the analysis based on AIC/BIC criteria. In the notation below, Y_{ij} is SEA seropositivity, A_{ij} is age, and S_{ij} is sampling year for child j in community i. D_i is distance from Lake Victoria to community i. Community-level random effects, b_i, were included in all models to allow for correlated outcomes within community. Functions $g(\cdot)$ were parameterized with cubic splines. Created with notebook https://osf.io/dnckx/.

**Model**	**AIC**	**BIC**
1. Exponential, constant rate model<br>$\log-\log\left[1-P\left(Y_{ij}=1\middle|A_{ij},b_i\right)\right]=\log\lambda+\log A_{ij}+b_i$	3730	3912
2. Semi-parametric proportional hazards model<br>$\log-\log\left[1-P\left(Y_{ij}=1\middle|A_{ij},b_i\right)\right]=g\left(A_{ij}\right)+b_i$	3697	3897
3. Semi-parametric proportional hazards model, controlling for survey year<br>$\log-\log\left[1-P\left(Y_{ij}=1\middle|A_{ij},S_{ij},b_i\right)\right]=g\left(A_{ij}\right)+\beta_2 I\left(S_{ij}=2013\right)+\beta_3 I\left(S_{ij}=2014\right)+b_i$	3692	3905
4. Semi-parametric proportional hazards model, controlling for survey year, including an indicator for communities <1.5km from the lake<br>$\log-\log\left[1-P\left(Y_{ij}=1\middle|A_{ij},D_i,S_{ij},b_i\right)\right]=g\left(A_{ij}\right)+\beta_1 I(D_i<1.5)+\beta_2 I\left(S_{ij}=2013\right)+\beta_3 I\left(S_{ij}=2014\right)+b_i$	3689	3886
5. Semi-parametric hazard model allowing for different baseline hazards among communities <1.5km from the lake versus further, controlling for survey year<br>$\log-\log\left[1-P\left(Y_{ij}=1\middle|A_{ij},D_i,S_{ij},b_i\right)\right]=g_1\left(A_{ij}\right)I(D_i<1.5)+g_2\left(A_{ij}\right)I(D_i\geq 1.5)+\beta_2 I\left(S_{ij}=2013\right)+\beta_3 I\left(S_{ij}=2014\right)+b_i$	3692	3900

* AIC: Akaike Information Criterion; BIC: Bayesian Information Criterion
